# Development and Morphometrics of the Turkestan Cockroach (*Periplaneta lateralis*) Under Solitary and Cohort Rearing at 25 and 30 °C

**DOI:** 10.3390/insects17090948

**Published:** 2026-09-11

**Authors:** Isha Budha Magar, Kristen Bowers, Paulina Quezada, Caleb B. Hubbard

**Affiliations:** Department of Entomology, Plant Pathology, and Weed Science, New Mexico State University, Las Cruces, NM 88003, USA

**Keywords:** peridomestic pest, stadium duration, developmental time, solitary rearing, cohort rearing, morphometrics

## Abstract

The Turkestan cockroach, *Periplaneta lateralis* (Walker), is an invasive peridomestic pest expanding across southwestern U.S. habitats, where it has been reported to replace the Oriental cockroach, *Blatta orientalis* L. Life-history advantages may contribute to this expansion, but its developmental parameters remain poorly characterized. We measured development at 25 and 30 °C under solitary rearing, solitary rearing with photographic monitoring, and cohort rearing. Developmental time was significantly shorter under cohort than solitary rearing. Solitary-reared males and females required 219.8 ± 1.45 and 225.0 ± 2.07 d at 25 °C and 181.5 ± 2.51 and 190.2 ± 2.13 d at 30 °C, respectively. Cohort-reared males and females required 120.2 ± 7.43 and 112.4 ± 9.20 d at 25 °C and 76.5 ± 2.66 and 77.6 ± 1.30 d at 30 °C. Across the two solitary-rearing experiments, individuals completed 7–11 instars. Morphometric measurements increased across instars but overlapped among late instars, making it an unreliable trait to use to determine instar. Degree-day accumulation was consistent across temperatures under cohort rearing but not under solitary rearing. Because the rearing protocols differed in housing, handling, monitoring, and incubator conditions, the difference cannot be attributed solely to social contact. These results demonstrate that laboratory rearing conditions should be considered when extrapolating laboratory estimates to field populations and when comparing laboratory strains with field strains.

## Simple Summary

The Turkestan cockroach, *Periplaneta lateralis* (Walker), is a common peridomestic pest found in habitats such as feed mills, animal barns, water meter boxes, and electrical boxes. Little research exists on the life history of this species, including how many developmental stages there are and how long each developmental stage takes to complete. We reared cockroaches alone or in cohorts at 25 and 30 °C and followed their development from newly hatched nymphs to adults. Cockroaches raised in cohorts reached adulthood in approximately eleven weeks at 30 °C and sixteen to seventeen weeks at 25 °C, compared with roughly six to seven months for those raised alone. Accumulated degree-day totals (heat units accumulated above an assumed 15 °C developmental threshold) under cohort rearing were similar across temperatures but differed significantly under solitary rearing conditions. These results provide a unique look into the development of Turkestan cockroaches and show that laboratory rearing methods strongly influence estimates used to predict pest development. The rapid development observed, which depends on rearing conditions, may contribute to how the Turkestan cockroach has spread rapidly across the southwestern United States and displaced the Oriental cockroach from outdoor habitats.

## 1. Introduction

Turkestan cockroaches (*Periplaneta lateralis* Walker, Blattodea: Blattidae) are an invasive peri-urban pest established across landscapes in the western United States [1]. First detected at military installations in California and Texas in 1978–1979, they are now distributed across much of the southern United States, where they are displacing the Oriental cockroach, *Blatta orientalis* L., as the dominant outdoor species [1,2,3]. Turkestan cockroaches thrive in warm, arid environments, exploiting sites with an abundance of organic matter (such as leaf debris and trash), animal feces in livestock facilities, irrigation systems, underground electrical boxes, feed mills, and water troughs [1,4]. Despite typically inhabiting peridomestic regions, they may occasionally enter human interior spaces through gaps, crevices, and openings between doors and windows [5,6,7]. Turkestan cockroaches are sexually dimorphic: males are fully winged and brownish in color, while females are black with greatly reduced wings [8].

A limited number of studies have examined the life history of the Turkestan cockroach, and accurate data on the number of instars and the stadium duration of each instar remain scarce. The first study on this species’ life history was conducted in Japan in 2008 [9]. According to the abstract, individuals could molt into adults after the seventh instar, development could not be completed at temperatures below 20 °C, and the fastest development occurred at 30–33 °C, where individuals could reach adulthood in as few as 73 days. We were unable to access the full text of this study to verify all results, as it appears to have been published only as a conference proceeding abstract.

Building on this limited groundwork, the foundational research by Kim and Rust [1] can be considered the only study to have examined the life history of these pests extensively. Using a Turkestan cockroach strain from California, they documented that Turkestan cockroaches needed a total of five nymphal instars to reach adulthood, with mean nymphal developmental periods of 222 days for males and 224 days for females when raised in cohorts at 26.7 ± 2 °C, which directly contradicts the seven instars documented by Imai [9].

Beyond temperature, other environmental factors also appear to influence development rates of Turkestan cockroaches. For example, Turkestan cockroaches developed faster when exposed to artificial lights and showed a slower growth rate under direct sunlight [10], which may explain their success in places like animal barns, where artificial lighting is abundant.

Beyond these few studies, no other research has examined the life history of this invasive pest, leaving several questions unanswered, including the stadium duration at each instar and the possibility of a variable number of instars, as has been documented in other peridomestic species such as the American cockroach (*Periplaneta americana* L.), the Asian cockroach (*Blattella asahinai* Mizukubo), and the Oriental cockroach (*Blatta orientalis*) [11,12,13]. In the present study, we conducted three experiments to characterize the development of *P. lateralis*. The first objective was to determine the number of instars required for the Turkestan cockroach to reach adulthood, the stadium duration of each instar, and instar-specific morphometry at 25 and 30 °C under solitary rearing. The second was to establish whether time-lapse photographic monitoring recovered molts that daily inspection could miss, using a separate set of individually housed nymphs at 30 °C. The third was to compare developmental time between solitary and cohort rearing at both temperatures. We hypothesized that the Turkestan cockroach would (i) develop faster at 30 °C than at 25 °C, (ii) accumulate a similar number of degree-days (DD) across both temperatures, and (iii) develop faster under cohort rearing than in solitary conditions. Definitive protocol-specific developmental data across temperature treatments will improve our understanding of laboratory variation seen in this species and provide a foundation for future field validation and pest-management models.

## 2. Materials and Methods

### 2.1. Cockroach Colony

For all studies, we used the “Las Cruces strain” of *P. lateralis*, which was originally collected in 2017 from the backyard of a residence with a chicken coop in Las Cruces, New Mexico, and has since been maintained at New Mexico State University. Colonies were reared in 15-gal plastic containers (IRIS USA Inc., Surprise, AZ, USA; Cat. No. 394008) at 26 ± 3 °C, 30–50% RH, and a 12:12 (L:D) h photoperiod, with ad libitum food (equal mixture of dog food (Post Consumer brand, Lakeville, MN, USA), cat food (J.M. Smucker Company, Orrville, OH, USA), and rabbit food (Kent Nutrition Group, Muscatine, IA, USA) mixed and ground into a fine powder) and water.

#### 2.1.1. Experiment 1: Life-History Traits Under Solitary Conditions at 25 and 30 °C

A completely randomized design with two temperature treatments (25 and 30 °C) was used to characterize the solitary life-history traits of the Turkestan cockroach. The temperatures were selected to extend the limited published life-history data for this species: 25 °C approximates the 26.7 ± 2 °C conditions used by Kim and Rust [1], whereas 30 °C falls within the range in which Imai [9] reported accelerated nymphal development. For each temperature, 250 freshly emerged, one-day-old nymphs were individually housed in tapered round plastic cups (Item No. 325PC, Dart Container Corporation, Mason, MI) and provided with ad libitum food and water (Figure S1). Food was placed in a 1.5 cm-diameter plastic cap (Wheaton/DWK Life Sciences, USA, Millville, NJ, USA; Cat. No. 225129), and water was supplied in a 4 mL plastic vial (Wheaton/DWK Life Sciences, Millville, NJ, USA; Cat. No. 242815) plugged with cotton. Each individually housed cockroach was covered with a ventilated mesh lid and placed in an environmental chamber (Thermo Fisher Scientific, Marietta, OH, USA; Model 3759) corresponding to its assigned temperature treatment (25 or 30 °C). Individual cockroaches were examined daily, and the date of each molt was recorded. For each instar, head width, pronotum width, pronotum length, and body length were measured in the first 50 individuals; for adults, 25 males and 25 females were measured separately. Measurements were taken after briefly anesthetizing the insects with CO_2_ (≤10 s) and measuring them under a Leica S6 D Greenough stereo microscope with 6.3:1 zoom (Leica Microsystems, Singapore; Item No. 10446297) using LAS (Leica Application Suite) version 4.12 for real-time imaging. Following methods described by Kim and Rust [1], head width was measured between the outermost margins of the eyes, pronotum width at its widest point, and pronotum length along the dorsal midline. Body length was measured from the vertex of the head to the tip of the abdomen in nymphs and adult females, and from the vertex to the tip of the wings in adult males. After measurements, cockroaches were returned to their respective containers, and any adverse events (death or escape) of nymphs before reaching adulthood were recorded. Sex was recorded at the imaginal molt; nymphs that did not reach adulthood were not sexed. Morphometry therefore required repeated CO_2_ anesthesia of the same individuals in later instars. A single environmental chamber was used per temperature treatment, held at 30–50% relative humidity. CO_2_ anesthesia has been shown to increase instar number and stadium duration in *Blattella germanica* [14]; exposure was therefore limited to ≤10 s per event and applied only when an individual was measured.

#### 2.1.2. Experiment 2: Photographic Monitoring of Individual Cockroach Development at 30 °C

A completely randomized design was used, with four replicates of 16 one-day-old first-instar nymphs. Nymphs were individually housed in 5.5-oz plastic cups (Great Value, Walmart Inc., Bentonville, AR, USA; Cat. No. 13510357513), provided ad libitum access to food and water, and housed in an environmental chamber (Thermo Fisher Scientific, Marietta, OH, USA; Cat. No. 3759) maintained at 30 °C under a 12:12 (L:D) h photoperiod. Each replicate was recorded using a 1080p day-and-night-vision Arducam USB camera (Arducam Technology Co., Ltd., Nanjing, China) mounted 55 cm above the platform. All four cameras were connected to a shared USB hub, which was connected to a laptop located outside the incubator (Figure S2). A Python script (Version 3.12) captured images at 30-min intervals throughout development, from the first instar to adulthood. Sixteen cups were evenly spaced on a 31 × 31 cm cardboard platform with cutouts that secured the cups during handling and maintained consistent positioning among images. Experiment 2 was designed to reduce the risk of undetected molts in Experiment 1, in which molting was confirmed by daily inspection alone. Cockroaches consume their exuviae shortly after ecdysis, and *Periplaneta americana* recovers more than half of the nitrogen contained in the shed cuticle [15], so a molt can pass unrecorded if the exuviae are eaten between inspections. The 30-min imaging interval allowed newly shed exuviae to be identified before they were consumed. In addition to photographic monitoring, each instar was marked with a distinct color on the thorax using oil-based Posca pens (Mitsubishi Pencil Company, Limited, Tokyo, Japan). After each molt, nymphs were briefly anesthetized with CO_2_ (≤10 s) and re-marked with the color corresponding to the new instar (first instar: black; second: pink; third: green; fourth: yellow; fifth: blue; etc.). Molting was observed daily by checking whether the marked color on the thorax had been shed, with further confirmation obtained from images taken the previous day. The four camera platforms constituted four independent replicates, each holding 16 individually housed nymphs in a 4 × 4 grid. Platform was included as a random effect in all Experiment 2 analyses. Experiment 2 was conducted at 30 °C only because available incubator capacity and the study timeline did not permit a parallel 25 °C photographic-monitoring treatment. This single-temperature scope is acknowledged in the Limitations.

#### 2.1.3. Experiment 3: Life-History Traits Under Cohort-Rearing Conditions at 25 and 30 °C

A completely randomized design with two temperature treatments was used to evaluate life-history traits under cohort-rearing conditions. At each temperature (25 or 30 °C), 1-d-old first-instar nymphs were divided into five replicate cohorts of 50 individuals and housed in plastic rearing containers (Sterilite Corporation, Townsend, MA, USA; Cat. No. 1892). Each container was provisioned with food, water, and harborage made from sections of molded-fiber egg cartons (Uline, Pleasant Prairie, WI, USA; Cat. No. S-25407). Food was provided ad libitum in 30-mm-diameter polypropylene lids (Thermo Scientific Nalgene, Rochester, NY, USA; Cat. No. DS3111-0030) and water was supplied ad libitum in 8-oz clear polyethylene cylinder bottles (Item No. S-18121, Uline, Pleasant Prairie, WI, USA) fitted with cellulose wicks cut from two-ply paper towels (Uline, Pleasant Prairie, WI, USA; Cat. No. S-7711). Each temperature treatment comprised five independent replicate containers of 50 nymphs (*k* = 5 containers, *n* = 250 individuals per temperature), with containers treated as the experimental unit in all analyses of cohort rearing. Individual molts were not recorded under cohort rearing because individuals were not uniquely marked and cast exuviae are rapidly consumed by conspecifics, preventing reliable assignment of molts to individual nymphs [15]. Individually marking all nymphs would also have required repeated handling and anesthesia, altering the otherwise low-disturbance cohort protocol.

Rearing containers were maintained in incubators (Quincy Lab Inc., Burr Ridge, IL, USA; Model 12-140E) set to the assigned temperature treatments. Food and water were replenished every 3 d, and containers were otherwise left undisturbed. During each check, dead nymphs were recorded and removed. After the first adult emerged, all containers were examined daily, and newly eclosed adults were counted and recorded.

### 2.2. Statistical Analysis

All statistical analyses were conducted in R version 4.4.2 using RStudio version 2026.01.0+392. Figures were generated using ggplot2, and mixed-effects models were fitted with the nlme package. All tests were two-sided, with α = 0.05. Analyses of developmental time to adulthood were conditional on reaching adulthood, whereas per-instar stadium-duration summaries included all recorded stadia, regardless of whether the individual survived to adulthood. One environmental chamber was used per temperature treatment in Experiments 1 and 2, and one incubator was used per temperature in Experiment 3, meaning chamber effects cannot be separated from temperature effects.

#### 2.2.1. Experiment 1: Solitary Rearing at 25 and 30 °C

Developmental time to adulthood was compared among the four temperature-by-sex groups using a Kruskal–Wallis test. Four prespecified pairwise Mann–Whitney U comparisons were then conducted: temperatures were compared within each sex, and sexes were compared within each temperature. Bonferroni correction was applied across these four comparisons (Results Section 3.1). For each instar, stadium duration was compared between 25 and 30 °C using a Wilcoxon rank-sum test, with Bonferroni correction applied across the ten instars (Results Section 3.1). The observed cumulative proportion of the original 250 nymphs per temperature reaching adulthood was plotted over time (Results Section 3.1), and mutually exclusive final outcomes were summarized as adulthood, natural death, mold-related loss, or escape/disappearance (Results Section 3.1).

For the morphometric measurements, a two-factor aligned rank transform (ART) ANOVA was used, with temperature (25 and 30 °C), developmental stage, and their interaction as fixed effects. Following significant interactions, simple-effect comparisons among developmental stages within each temperature were obtained using estimated marginal means (emmeans) with Holm adjustment. To provide direct between-temperature comparisons within each developmental stage and adult sex, we also used Welch *t*-tests with Holm correction across the 48 contrasts. The morphometric table summary presents compact-letter groupings for the ART/emmeans stage comparisons and asterisks for the between-temperature contrasts; full Welch test statistics are reported in Table S3. Statistical significance was set at α = 0.05.

#### 2.2.2. Experiment 2: Photographic Monitoring of Individual Cockroaches at 30 °C

Developmental time and the number of instars were compared among the four camera platforms. We also calculated the intraclass correlation coefficient (ICC) and design effect to determine how much of the variation in developmental time was associated with differences among platforms. Adult-only stadium durations by instar were summarized in Results Section 3.2. 

##### Comparisons of Stadium Duration in Experiments 1 and 2 at 30 °C

Stadium duration was compared between the two solitary-rearing protocols at 30 °C. Because each cockroach contributed stadium-duration measurements for multiple instars, we used a mixed-effects model to account for repeated measurements from the same individual. Stadium duration was log-transformed, with experiment, instar, and their interaction included as fixed effects and individual cockroach included as a random intercept. Overall effects were evaluated using *F* tests. Experiments 1 and 2 were then compared separately at each instar, with Bonferroni correction applied across the ten comparisons (Results Section 3.2). As a secondary analysis, Experiments 1 and 2 were also compared at each instar using Wilcoxon rank-sum tests to determine whether the conclusions were similar when using a different statistical approach. Bonferroni correction was applied across the ten comparisons (Table S2).

#### 2.2.3. Experiment 3: Cohort Rearing at 25 and 30 °C

The rearing container was treated as the experimental unit because nymphs housed within the same container were not statistically independent. Developmental time was analyzed using a linear mixed-effects model fitted to log-transformed days, with temperature, sex, and their interaction as fixed effects and container nested within temperature as a random intercept. Marginal F tests were used to evaluate the fixed effects. Male-versus-female contrasts were obtained within each temperature from estimated marginal means, with Bonferroni correction applied across the two sex contrasts. Means and SEMs were calculated from the five sex-specific container means at each temperature, whereas *n* and ranges describe individual adults. Developmental-time contrasts were also summarized descriptively across selected quantiles of the individual-level distribution.

#### 2.2.4. Comparison of Solitary (Experiment 1) and Cohort (Experiment 3) Rearing

Developmental time under solitary and cohort rearing was compared separately for each sex and temperature at the experimental-unit level. Individual cockroaches were treated as experimental units under solitary rearing, whereas sex-specific container means were treated as experimental units under cohort rearing. Log-transformed developmental times were compared using Welch tests, with Bonferroni correction applied across the four rearing-condition contrasts. Ratios of geometric mean developmental time under solitary relative to cohort rearing and their 95% confidence intervals were reported as effect estimates. As a rank-based sensitivity analysis, Mann–Whitney U tests and signed-rank biserial correlations with bootstrap 95% confidence intervals were calculated using the same experimental-unit-level observations, with Bonferroni correction across the four comparisons (Figure S4).

##### Effect Sizes

Signed rank-biserial correlations were calculated from Mann–Whitney U statistics. Absolute values were classified as small (|*r*| < 0.30), medium (0.30 ≤ |*r*| < 0.50), or large (|*r*| ≥ 0.50). Bootstrap confidence intervals were obtained by resampling the applicable experimental unit: individual cockroaches for solitary rearing and sex-specific container means for cohort rearing.

##### Degree-Day Calculations

Thermal accumulation was estimated using degree-day (DD) values, the cumulative physiological heat units required to complete a developmental stage [16]. Under fluctuating field temperatures, degree-days are commonly calculated from daily maximum and minimum temperatures relative to a lower developmental threshold. Because these experiments were conducted at constant temperatures, thermal accumulation was calculated as DD = (T − T_0_) × d, where T is the constant rearing temperature (25 or 30 °C), T_0_ is the assumed lower developmental threshold (15 °C), and d is the number of calendar days from first-instar nymph to adult eclosion. Degree-day values are reported in Section 3.1. A 15 °C threshold was selected because it falls within the range reported for *Blattella asahinai* and *Blattella germanica* [17,18]. Published thresholds identified for Periplaneta include 17.1 °C for first-instar *P. australasiae* [19] and 6.8 °C for *P. americana* embryos [20], neither directly applicable to the present study. The lower developmental threshold was not estimated by linear regression because only two rearing temperatures were evaluated. 

## 3. Results

### 3.1. Experiment 1: Life-History Traits Under Solitary Conditions at 25 and 30 °C

**Figure 1:**
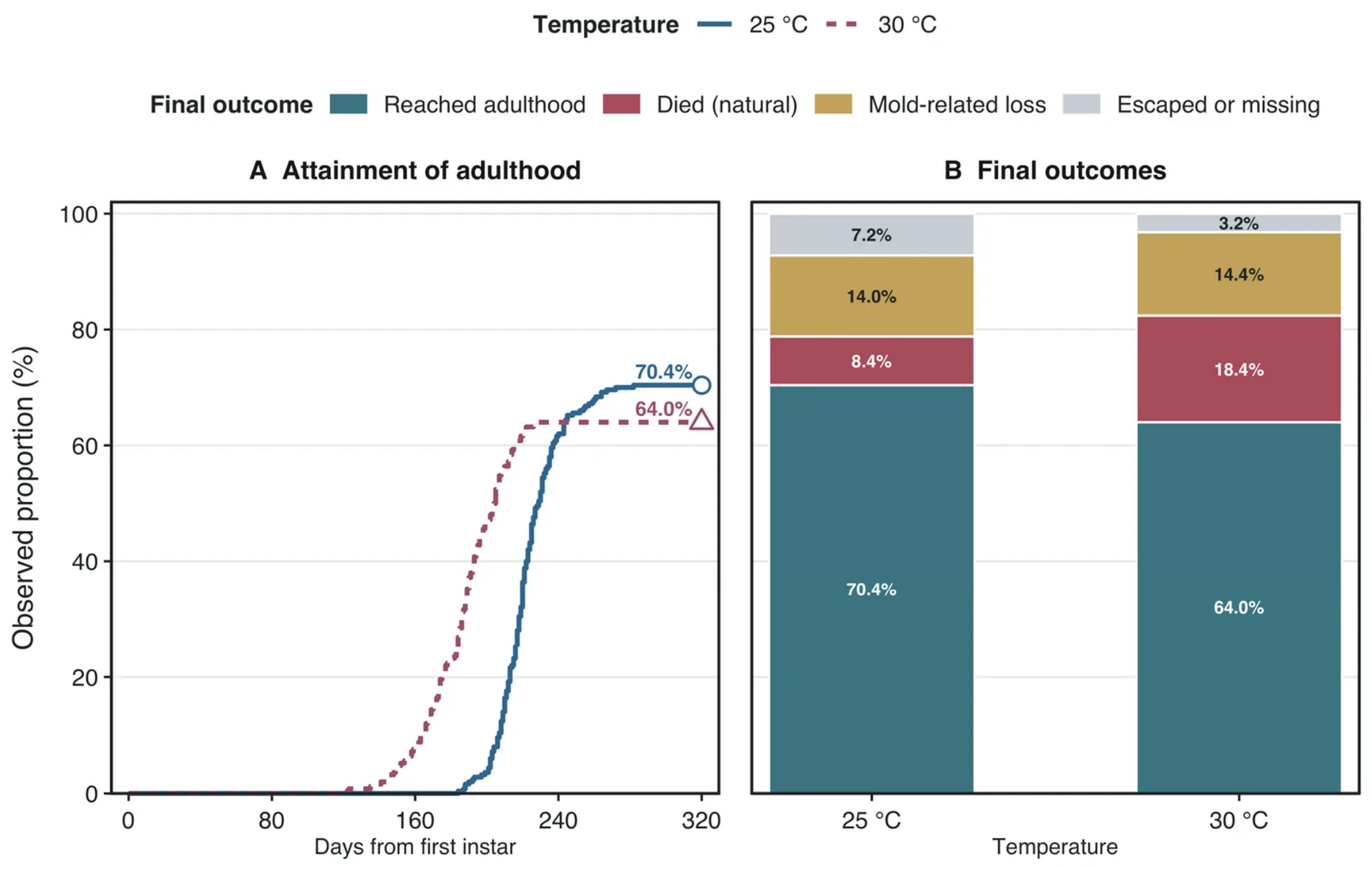
Adult emergence and final outcomes under solitary rearing at 25 and 30 °C (Experiment 1). (**A**) shows the cumulative percentage of the original 250 nymphs at each temperature that reached adulthood (blue circle for 25 °C and red triangle for 30 °C are the endpoint markers highlighting final values). (**B**) shows the final percentage reaching adulthood or lost through natural death, mold, or escape. All 500 nymphs were included.

Of the 250 nymphs established at each temperature, 176 (70.4%) reached adulthood at 25 °C and 160 (64.0%) reached adulthood at 30 °C. Adults at 25 °C were 52.8% male and 47.2% female, whereas adults at 30 °C were 52.5% male and 47.5% female. Expressed as percentages of the 250 nymphs established at each temperature—the same denominator used in Figure 1B—losses at 25 °C comprised 8.4% natural death, 14.0% mold-related loss, and 7.2% escape (21, 35, and 18 individuals, respectively), and losses at 30 °C comprised 18.4% natural death, 14.4% mold-related loss, and 3.2% escape (46, 36, and 8 individuals, respectively). Final outcomes are summarized in Figure 1B. During the experiment, mold was associated primarily with water-container leakage and reduced airflow where containers were stacked in the incubators.

Developmental time from the first instar to adulthood differed significantly among the four temperature-by-sex groups (*H* = 183.83, df = 3, *p* < 0.001). Both males and females developed more slowly at 25 °C than at 30 °C (males: adjusted *p* < 0.001, |*r*| = 0.870; females: adjusted *p* < 0.001, |*r*| = 0.838). Mean developmental time at 25 °C was 219.8 ± 1.45 d for males and 225.0 ± 2.07 d for females, compared with 181.5 ± 2.51 d and 190.2 ± 2.13 d, respectively, at 30 °C (Table 1). Developmental time did not differ significantly between males and females within either temperature after correction for multiple comparisons (Table 2).

Consistent with the shorter developmental times at 30 °C, developmental rates increased from 4.55 × 10^−3^ d^−1^ for males and 4.44 × 10^−3^ d^−1^ for females at 25 °C to 5.51 × 10^−3^ d^−1^ and 5.26 × 10^−3^ d^−1^, respectively, at 30 °C. These values represented increases of 21% for males and 18% for females (Figure S3).

**Figure 2:**
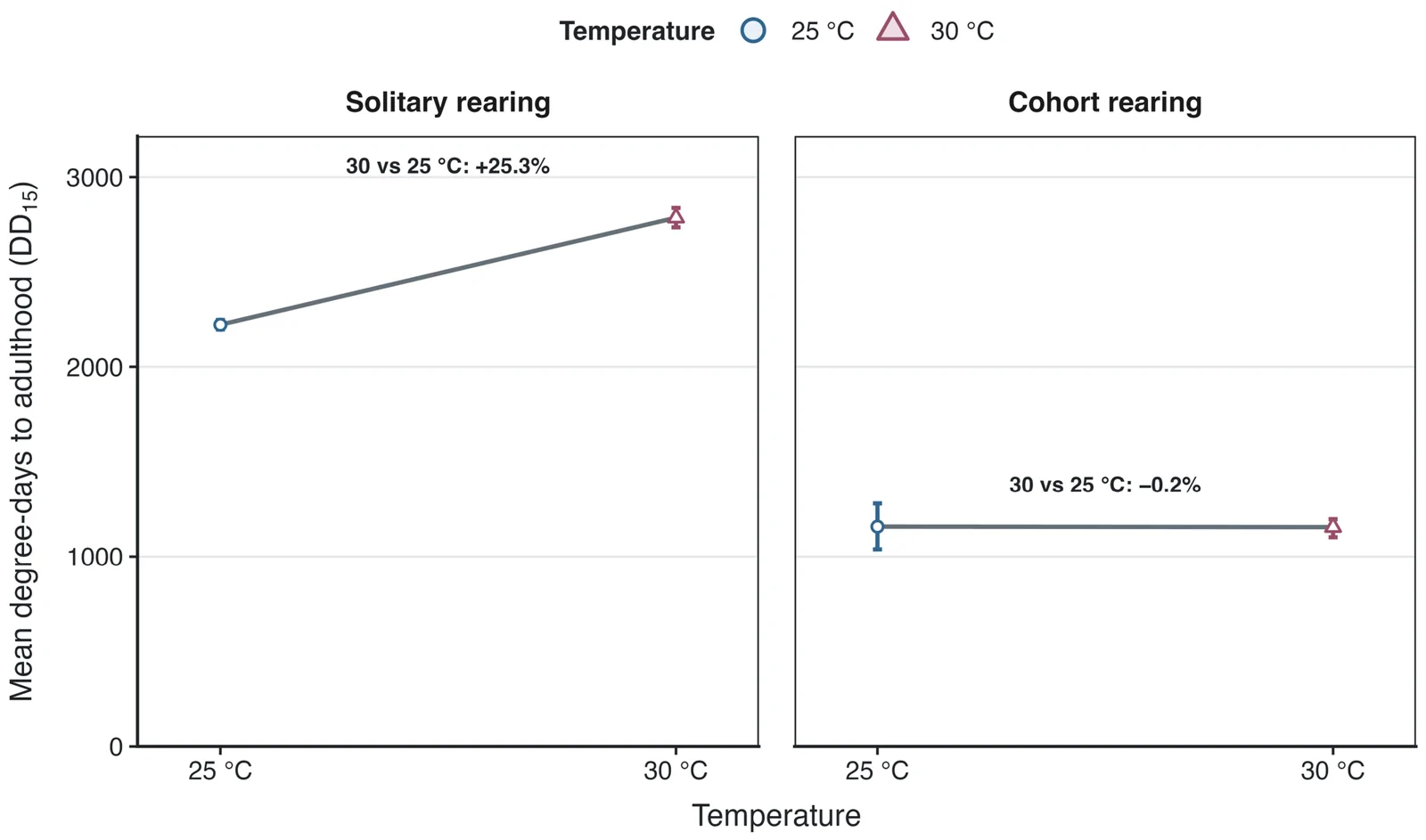
Degree-day accumulation from first instar to adulthood under solitary and cohort rearing at 25 and 30 °C (Experiments 1 and 3). Points represent mean degree-day accumulation ± 95% confidence intervals.

Accumulated degree-days to adulthood at 25 °C were 2198 ± 14.5 DD for males and 2250 ± 20.7 DD for females. At 30 °C, degree-day accumulation was higher, reaching 2722 ± 37.6 DD for males and 2853 ± 31.9 DD for females (Figure 2; Table 1). The differences between temperatures were approximately 524 DD for males and 603 DD for females.

**Figure 3:**
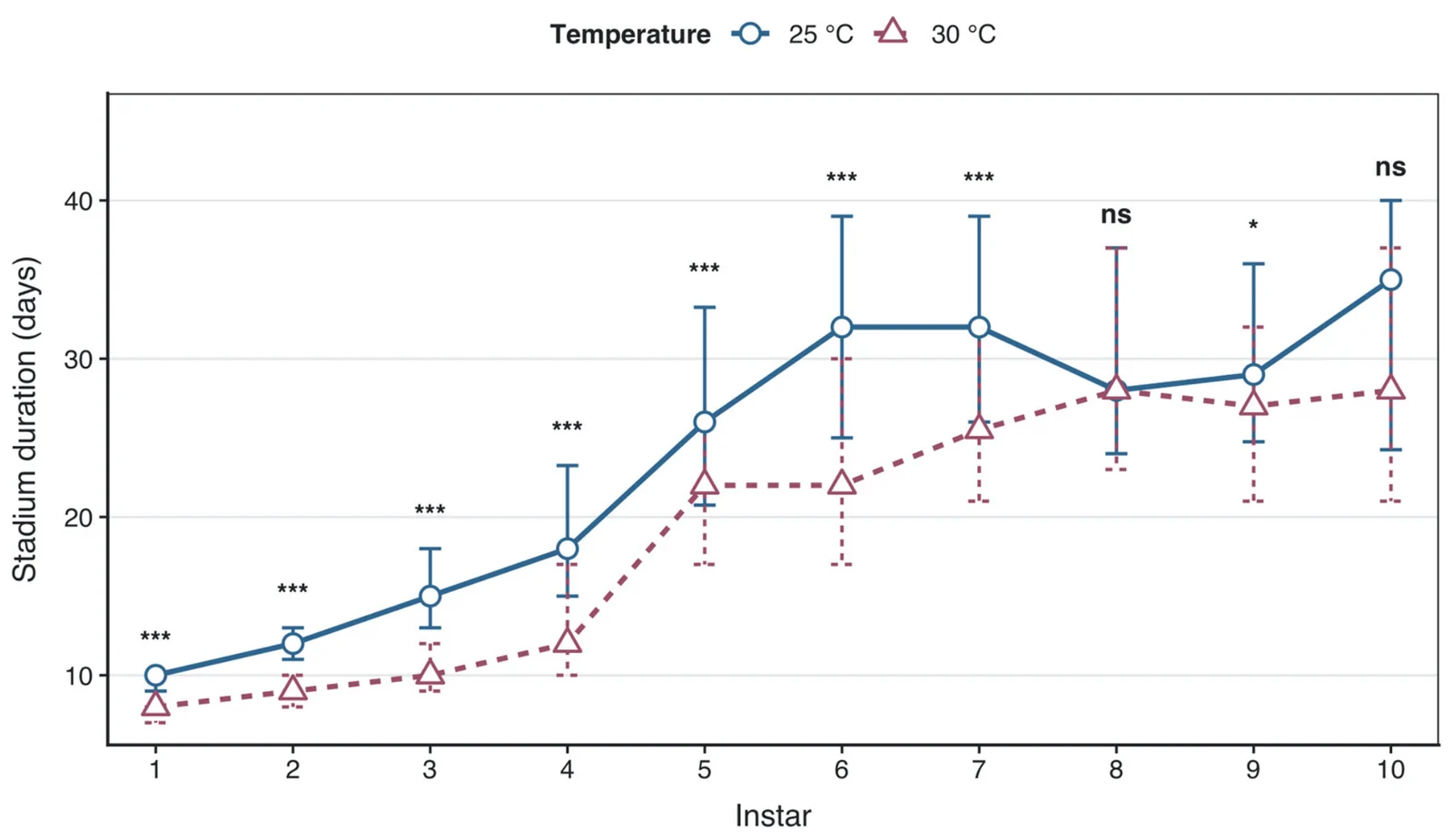
Stadium duration under solitary rearing at 25 and 30 °C (Experiment 1). Points show median stadium duration, and error bars show the interquartile range. Only individuals that reached adulthood were included. Significance levels are defined as follows: ns = non-significant (*p* > 0.05), * = *p* < 0.05, and *** = *p* < 0.001. Statistical comparisons using all recorded stadia are presented in Table 4.

Across both temperatures, most adults completed either nine (49.1%) or ten instars (37.8%) (Table 3; Table S1). The number of instars required to reach adulthood ranged from seven to ten at 25 °C and from eight to ten at 30 °C. Stadium duration was significantly longer at 25 °C than at 30 °C for instars 1–7 and 9 after correction for multiple comparisons, whereas no significant temperature differences were detected for instars 8 or 10 (Figure 3; Table 4). The first instar had the shortest mean duration at both temperatures: 10.01 ± 0.09 d at 25 °C and 7.77 ± 0.07 d at 30 °C, corresponding to 100.1 ± 0.91 and 116.6 ± 1.12 DD, respectively. The longest mean stadium duration occurred during the tenth instar at 25 °C (33.76 ± 1.28 d; 337.6 ± 12.83 DD) and the eighth instar at 30 °C (30.21 ± 0.69 d; 453.2 ± 10.32 DD).

The morphometric analysis showed a significant effect of developmental stage for all four traits: head width (*F*_(11, 1076)_ = 2038.719, *p* < 2.22 × 10^−16^), pronotum width (*F*_(11, 1076)_ = 1837.176, *p* < 2.22 × 10^−16^), pronotum length (*F*_(11, 1076)_ = 1758.356, *p* < 2.0 × 10^−16^), and body length (*F*_(11, 1076)_ = 4050.194, *p* < 2.22 × 10^−16^). The overall temperature effect was significant for head width (*F*_(1, 1076)_ = 17.507, *p* = 3.10 × 10^−5^), pronotum width (*F*_(1, 1076)_ = 55.899, *p* = 1.58 × 10^−13^), and body length (*F*_(1, 1076)_ = 10.140, *p* = 0.00149), but not pronotum length (*F*_(1, 1076)_ = 0.469, *p* = 0.493). Temperature × developmental-stage interactions were significant for head width (*F*_(11, 1076)_ = 5.632, *p* = 7.53 × 10^−9^), pronotum width (*F*_(11, 1076)_ = 6.694, *p* = 6.27 × 10^−11^), pronotum length (*F*_(11, 1076)_ = 1.912, *p* = 0.0343), and body length (*F*_(11, 1076)_ = 4.663, *p* = 5.48 × 10^−7^). Holm-adjusted stage groupings are shown in Table 5. Measurements generally increased with developmental stage, but ranges overlapped among several adjacent late instars, limiting the reliability of any single measurement for instar assignment. In the separate between-temperature analysis, seven of 48 Welch/Holm contrasts were significant: pronotum width at instars 2, 4, 7, and 8; head width at instar 4; and body length at instars 8 and 9. The 25 °C mean was larger in each significant contrast, and no adult trait differed significantly between temperatures (Table 5; Table S3).

### 3.2. Experiment 2: Photographic Monitoring of Individual Cockroaches at 30 °C

Of the 64 nymphs reared individually under time-lapse photographic monitoring at 30 °C, 52 (81.3%) reached adulthood. Five individuals died (7.8%), four were lost or unaccounted for (6.3%), and three had not completed the final molt to adulthood by the administrative endpoint on 14 July 2026 and were retained as right-censored observations. Most losses occurred during the early instars.

Adults were obtained from all four camera platforms; however, developmental time differed significantly among platforms, with mean values of 167.9, 212.2, 280.6, and 294.5 d (*H* = 26.48, *p* < 0.001). The number of instars required to reach adulthood also differed among platforms (*p* < 0.001), with individuals completing between eight and eleven instars. A substantial proportion of the variation in developmental time was attributable to differences among camera platforms (ICC = 0.62; design effect = 8.36).

The first instar had the shortest mean stadium duration (8.31 ± 0.17 d), with stadium duration generally increasing across subsequent instars (Table 6). Mean developmental time from eclosion to the final molt to adulthood was 221.6 ± 13.94 d for males (*n* = 26, range 108–315 d) and 255.3 ± 13.24 d for females (*n* = 26, range 114–356 d). These developmental times corresponded to 3324 ± 209.1 DD for males and 3829 ± 198.6 DD for females, with ranges of 1620–4725 and 1710–5340 DD, respectively (Table 1).

**Figure 4:**
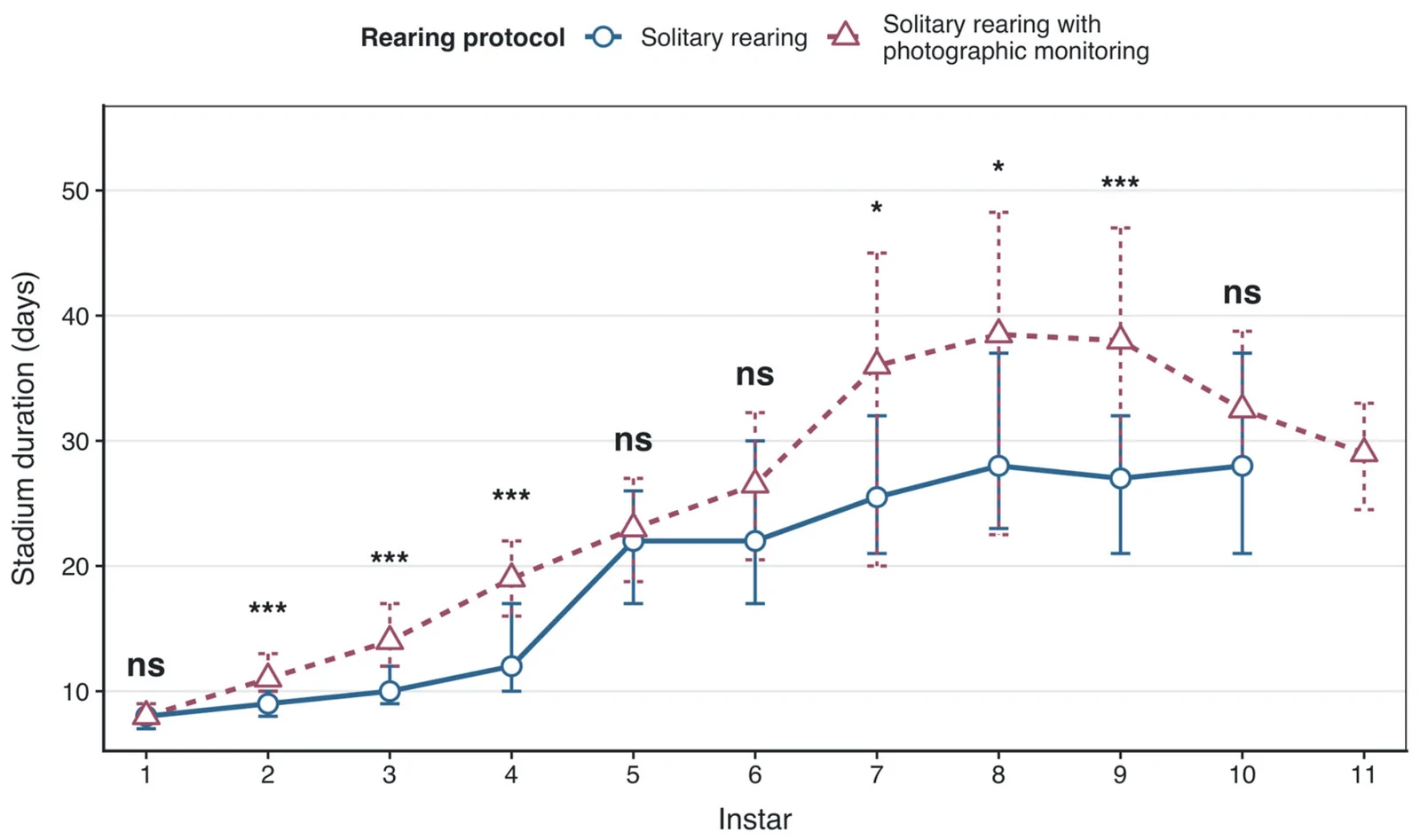
Stadium duration at 30 °C in Experiments 1 and 2. Points show median duration, and error bars show the interquartile range. Significance symbols are based on direct contrasts from the mixed-effects model, with Bonferroni correction across the ten instars. Only individuals that reached adulthood were included. *** adjusted *p* < 0.001; * adjusted *p* < 0.05; ns = not significant.

Mean total developmental time was greater in Experiment 2 than in Experiment 1 under the same solitary-rearing temperature of 30 °C. In Experiment 1, males and females reached adulthood in 181.5 ± 2.51 d and 190.2 ± 2.13 d, respectively. Individuals in Experiment 1 completed 8–10 instars, whereas those in Experiment 2 completed 8–11 instars, including an eleventh instar that was not observed in Experiment 1. Stadium duration differed between Experiments 1 and 2 overall (*F*_(1, 206)_ = 81.12, *p* < 0.001), and the significant experiment × instar interaction indicated that the difference between experiments varied among instars (*F*_(9, 1723)_ = 4.43, *p* < 0.001). Stadium duration was longer in Experiment 2 at instars 2 (ratio = 1.25, adjusted *p* < 0.001), 3 (ratio = 1.33, adjusted *p* < 0.001), 4 (ratio = 1.43, adjusted *p* < 0.001), 7 (ratio = 1.17, adjusted *p* = 0.030), 8 (ratio = 1.17, adjusted *p* = 0.036), and 9 (ratio = 1.29, adjusted *p* < 0.001). No significant differences were detected at instars 1, 5, 6, or 10 (Table 6; Figure 4). The Wilcoxon rank-sum analysis identified differences at instars 1–4 and 9 (Table S2).

The two analyses differ at instars 7 and 8 because the mixed model estimates instar-specific contrasts on the log scale with individual as a random intercept, drawing power from the repeated-measures structure, whereas the rank-sum tests treat each instar as an independent sample.

### 3.3. Experiment 3: Cohort Rearing at 25 and 30 °C

Across the five replicate containers at each temperature, 226 of 250 nymphs reached adulthood at 25 °C (90.4%; 112 males and 114 females), whereas 221 of 250 reached adulthood at 30 °C (88.4%; 109 males and 112 females).

At 25 °C, mean container-level developmental time was 120.2 ± 7.43 d for males and 112.4 ± 9.20 d for females, corresponding to 1201.7 ± 74.3 and 1124.0 ± 92.0 DD, respectively. At 30 °C, the corresponding means were 76.5 ± 2.66 d for males and 77.6 ± 1.30 d for females, corresponding to 1147.2 ± 39.9 and 1163.7 ± 19.5 DD, respectively. Individual developmental times ranged from 63 to 330 d for males and from 64 to 344 d for females at 25 °C, and from 50 to 118 d for males and from 51 to 121 d for females at 30 °C (Table 1).

Developmental time differed significantly between temperatures (*F*_(1, 8)_ = 22.01, *p* = 0.0016), but neither the overall effect of sex (*F*_(1, 435)_ = 1.47, *p* = 0.225) nor the temperature × sex interaction (*F*_(1, 435)_ = 1.00, *p* = 0.319) was significant. Although container-level mean developmental times differed numerically between the sexes at 25 °C, male and female developmental times did not differ significantly at either 25 °C (adjusted *p* = 0.451) or 30 °C (adjusted *p* = 1.000). These model results were based on 447 adults distributed among 10 containers.

**Figure 5:**
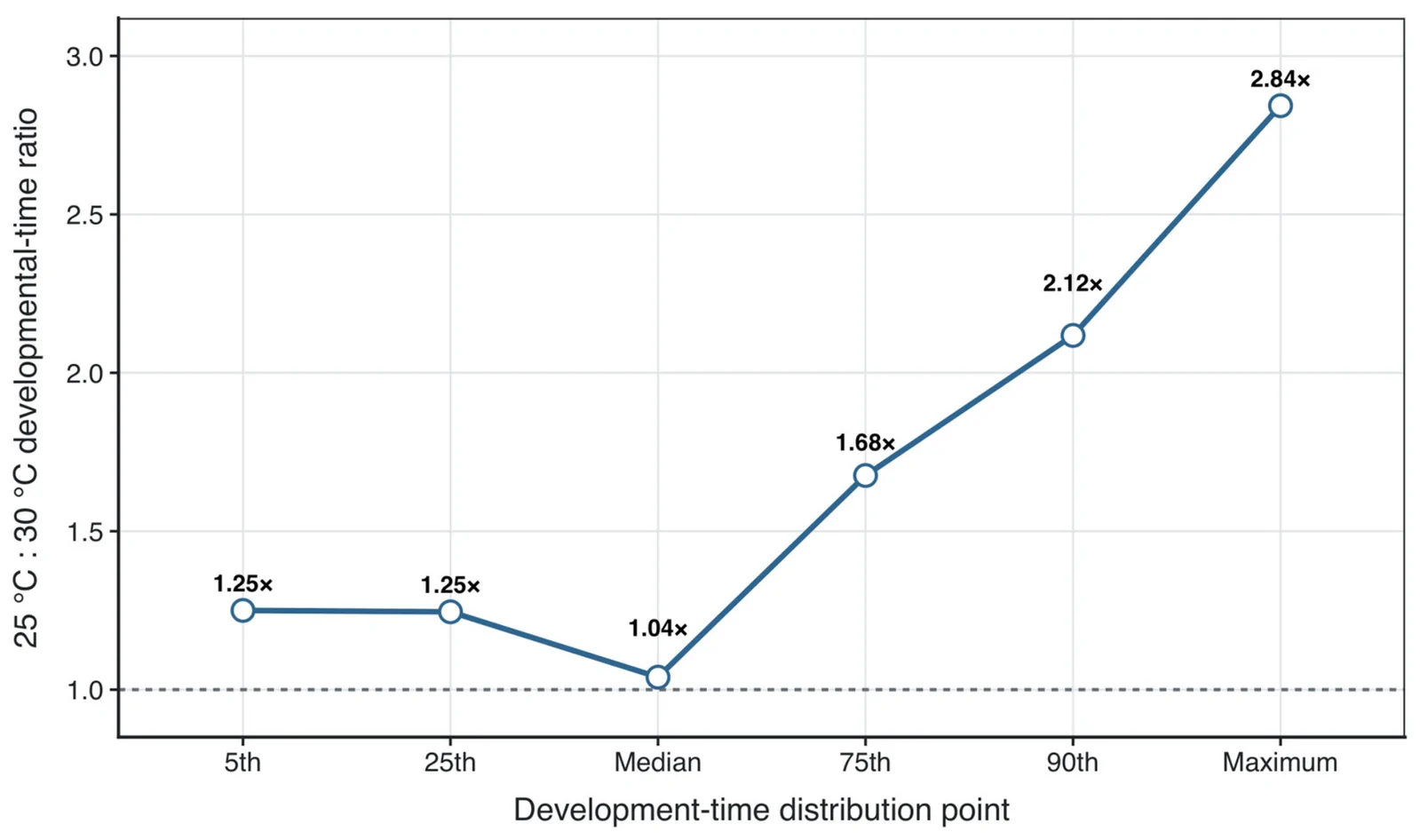
Comparison of developmental-time quantiles under cohort rearing at 25 and 30 °C. Points show the ratio of developmental time at 25 °C to that at 30 °C at selected percentiles and at the maximum. A ratio of 1 indicates equal developmental time. These individual-level quantiles are descriptive; formal temperature comparisons treated the container as the experimental unit.

Mean developmental time therefore differed significantly between temperatures, while the centers of the two distributions nearly coincided: at the individual level, median developmental times under cohort rearing were similar at 25 and 30 °C (79 and 76 d, respectively). However, the temperature difference was concentrated in the upper portion of the developmental-time distribution: the 90th-percentile developmental time at 25 °C was 2.12 times as long as that at 30 °C. Consistent with this pattern, 35.8% of adults at 25 °C required more than 110 d to complete development, compared with 4.5% at 30 °C (Figure 5).

### 3.4. Comparison of Solitary (Experiment 1) and Cohort (Experiment 3) Rearing

**Figure 6:**
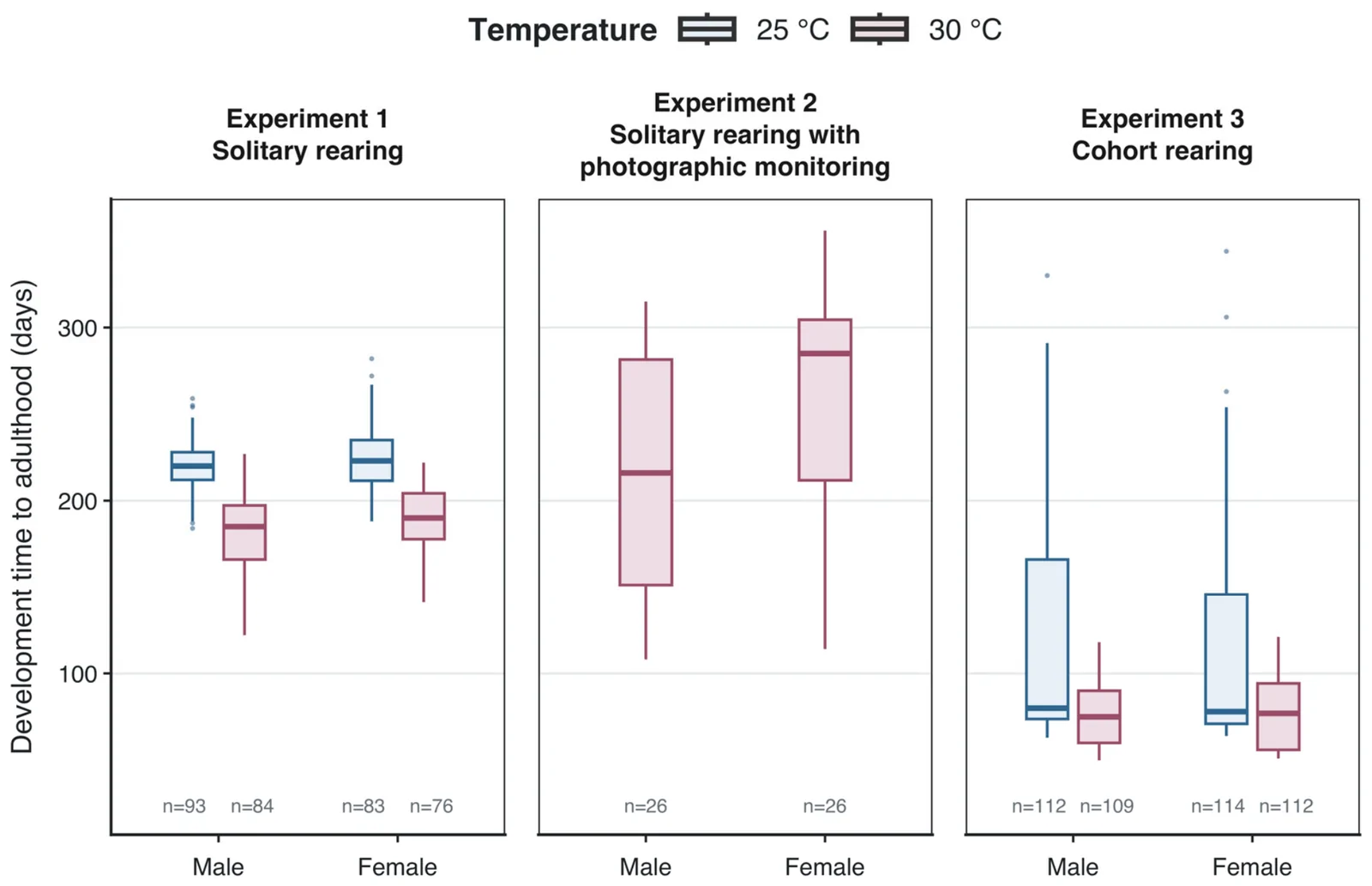
Developmental time from first instar to adulthood in male and female cockroaches under solitary rearing, solitary rearing with photographic monitoring, and cohort rearing. Boxes show the median and interquartile range, and whiskers extend to 1.5 times the interquartile range, with individual dots representing statistical outliers. Only individuals that reached adulthood were included.

Developmental time was significantly shorter under cohort rearing than under solitary rearing for both sexes at both temperatures (Figure 6). At 25 °C, geometric mean developmental time under solitary rearing was 1.84 times that under cohort rearing for males (95% CI: 1.54–2.21; adjusted *p* = 0.0027) and 2.02 times that under cohort rearing for females (95% CI: 1.63–2.51; adjusted *p* = 0.0031). At 30 °C, the corresponding ratios were 2.36 for males (95% CI: 2.14–2.60; adjusted *p* < 0.001) and 2.44 for females (95% CI: 2.33–2.56; adjusted *p* < 0.001) (Table 2).

At 30 °C, cohort rearing reduced arithmetic mean developmental time by 57.9% in males, from 181.5 ± 2.51 d under solitary rearing to 76.5 ± 2.66 d under cohort rearing, and by 59.2% in females, from 190.2 ± 2.13 d to 77.6 ± 1.30 d. Accumulated degree-days were likewise 57.9% lower for males and 59.2% lower for females under cohort rearing at 30 °C.

The rank-based analysis produced similar conclusions, with separation between solitary- and cohort-reared experiments for both sexes at both temperatures (all |*r*| = 1.00; adjusted *p* < 0.001; Figure S4).

## 4. Discussion

This study provides the first detailed stadium-duration and morphometric series for *P. lateralis* at 25 and 30 °C and shows that individually reared nymphs completed 7–11 instars before reaching adulthood. Most nymphs completed nine or ten instars before becoming adults, compared with the five reported for a California colony and the seven reported in Japan [1,9]. Variation in instar number is common among insects, including cockroaches, and may be influenced by sex, nutrition, injury, body-size thresholds, and rearing conditions [13,21,22,23]. Variable instar numbers have been reported in other cockroach species, including *Blattella asahinai*, which may complete six or seven instars [12], *Periplaneta americana*, which may complete 12–14 instars [11], and *Blattella germanica*, which commonly completes five or six instars [24]. Wild populations of *P. americana* have also been reported to complete more instars than laboratory populations, with 13–14 instars in wild females and 13 instars in wild males, compared with 12 instars in both sexes under laboratory conditions [11].

Nutritional stress and injury may contribute to variation in instar number. Under unfavorable conditions, insects may complete additional molts before reaching the body-size threshold required for metamorphosis [21]. Limited access to food or water has been associated with supernumerary molts in Manduca sexta [25] and *P. americana* [11].

Injury may also influence cockroach development and the number of instars completed. For example, cockroaches that regenerated injured legs with four-segmented tarsi, rather than the typical five-segmented tarsi, underwent more instars before reaching adulthood [22]. Interspecific differences in instar number may also be related to body size. Smaller species, such as *B. germanica* and *B. asahinai*, generally complete five to seven instars, whereas larger species, including *P. americana* and *P. australasiae*, may complete 10–13 instars [26].

Rearing context may also influence instar number. Short and Edwards reported that isolated *B. orientalis* females completed as many as 12 instars, compared with 8–10 under mixed-group conditions [13]. This pattern is consistent with the possibility that isolation contributed to the higher instar numbers observed in our study. However, because instar number was not recorded under cohort-rearing conditions, we could not directly compare solitary- and cohort-reared *P. lateralis* or determine whether isolation increased the number of instars completed.

Variation between the two solitary-rearing experiments further suggests that differences in experimental handling may also have influenced instar number. Individuals in Experiment 1 completed 7–10 instars, whereas those in Experiment 2 completed 8–11 instars, including an eleventh instar that was not observed in Experiment 1. In Experiment 2, individuals were anesthetized with CO_2_ and re-marked after every molt, whereas individuals in Experiment 1 were anesthetized only when selected for morphometric measurements. CO_2_ exposure, particularly during the early instars, has been reported to increase instar number and prolong stadium duration in *B. germanica* [14]. Although each exposure in our study lasted no more than 10 s, some individuals in Experiment 2 were exposed repeatedly as they progressed through successive instars. Repeated handling, marking, and CO_2_ exposure may have contributed to longer development and additional instars. The differences at instars 7–9 are also consistent with a cumulative procedural effect: by those stages, individuals in Experiment 2 had undergone repeated cycles of handling, CO_2_ anesthesia, and re-marking after each preceding molt, whereas Experiment 1 insects experienced less frequent handling. However, these procedural exposures remained confounded with other protocol differences, including incubator conditions, and cannot be assigned a causal contribution from the present design.

Despite variation between the solitary-rearing experiments, both produced substantially longer developmental times than the cohort-rearing protocol. Development was significantly shorter under cohort-rearing conditions at both temperatures, a pattern consistent with classic group effects in gregarious cockroaches [27,28]. Isolation has been shown to retard development throughout nymphal growth in *P. americana*, with subsequent experiments identifying physical contact as an important developmental stimulus [29,30]. Tactile stimulation has also been shown to accelerate development in grouped *B. germanica* and *Symploce pallens* (Stephens) [31]. However, the solitary- and cohort-rearing protocols also differed in container dimensions, handling, inspection frequency, incubator model, and CO_2_ exposure. Tactile stimulation itself has a long experimental history in cockroaches: crowding and its associated growth factors accelerate development in *Periplaneta americana* [29,30], and antennal contact has been proposed as the principal sensory channel through which these group effects operate. Our design cannot separate tactile stimulation from chemical or coprophagic cues among grouped individuals, and we therefore treat contact as one of several non-exclusive mechanisms.

Within this broader protocol-level effect, several biologically plausible mechanisms may have contributed to the shorter development observed under cohort-rearing conditions. Tactile stimulation from conspecifics and contact with body-supporting shelter are among the potential contributors to group effects in cockroaches. Shelter availability can influence nymphal development in *B. germanica* [32], and deprivation of body-contacting surfaces can prolong development [33]. Isolated nymphs in the present study lacked both direct contact with conspecifics and the shared harborage provided under cohort rearing. These differences may have contributed to the longer developmental times observed under solitary rearing.

In addition to these physical aspects of the rearing environment, cohort rearing may have provided greater exposure to frass and nestmate-associated microorganisms. *P. lateralis* harbors a diverse hindgut microbiota, and germ-free cockroach models show that environmental microbial exposure contributes to the establishment of the gut community [34,35,36]. In *P. americana*, preventing normal microbial acquisition prolonged development and impaired gut growth, whereas consumption of conspecific frass partially restored normal development [37,38]. Cohort-reared nymphs may therefore have received repeated microbial inoculation that was unavailable to isolated nymphs [39]. This possibility should be evaluated by independently manipulating frass exposure while holding shelter, density, food availability, and handling constant.

Beyond differences in microbial exposure, the number of individuals present may also influence developmental rate. Previous work with *P. americana* showed that isolation slowed development, but very high densities could also reduce survival and growth, with the fastest development occurring at an intermediate density [29,30]. Future studies should therefore evaluate a range of group sizes rather than comparing only isolated individuals with cohorts of 50. Such experiments should hold container area per insect, shelter, food availability, humidity, handling, and chamber assignment constant to separate the effect of density from other cues.

In addition to questions surrounding density, the cohort-rearing experiment revealed temperature-related differences in the distribution of developmental times. Individual-level median developmental times were similar at 25 and 30 °C, but the 25 °C treatment produced a substantially broader upper portion of the developmental-time distribution. The 90th-percentile developmental time at 25 °C was 2.12 times longer than at 30 °C, and 35.8% of adults at 25 °C required more than 110 days to complete development, compared with 4.5% at 30 °C. This longer upper range suggests that mean developmental time alone may not fully represent late-emerging individuals. Additional studies under fluctuating temperature conditions are needed to determine whether these laboratory findings can be used to inform monitoring intervals.

Previous laboratory studies provide additional context for the developmental times observed under controlled conditions. Despite differences in the number of instars completed, the developmental times observed in this study were generally consistent with previous reports. Under solitary rearing at 25 °C, males and females required 219.8 ± 1.45 and 225.0 ± 2.07 d to complete development, respectively. These values were similar to the 222 and 224 d reported by Kim and Rust at approximately 26.7 °C [1] and were also comparable to the 201 d reported by Imai at 25 °C [9]. Together, these findings suggest that similar total developmental times may result from differences in the number or duration of individual stadia. Variation in instar number among studies may reflect differences in colonies, rearing conditions, handling, or other factors that were not evaluated independently.

Rearing conditions also appeared to influence the degree-day estimates. Degree-day totals calculated using a 15 °C base were similar between temperatures under cohort rearing but differed significantly under solitary rearing, indicating that a single DD_15_ constant did not appropriately describe both solitary treatments.

In addition to developmental timing, morphometric measurements were evaluated as potential indicators of instar. Head width, pronotum width, pronotum length, and body length increased across successive instars, but measurements overlapped among several adjacent late instars. This overlap limits the reliability of assigning instar number based on a size measurement. Combining multiple body measurements with sex and the number of cercal annuli may improve noninvasive instar identification. Peterson et al. used pronotum measurements and cercal annuli to estimate instars in *B. asahinai* [12]. A similar approach for *P. lateralis* should be tested using individuals with known molt histories to determine how accurately it identifies each instar.

The differences between rearing protocols may also have broader implications for the life history of *P. lateralis*. Faster development under cohort-rearing conditions could provide an advantage in aggregated populations. However, this study did not evaluate development in the field, reproductive output, population growth, or competition with *B. orientalis*. The results therefore cannot explain the reported replacement of Oriental cockroaches in outdoor habitats, but they identify rapid development under cohort conditions as a potential contributing trait that should be examined through field studies and direct competition experiments.

### Limitations

Several limitations should be considered when interpreting these results. The solitary- and cohort-rearing experiments differed in several ways, including container type, harborage, handling, and chamber assignment. Because of these differences, we cannot attribute the observed developmental differences to social contact alone. Photographic monitoring in Experiment 2 was also conducted only at 30 °C due to limited incubator space, so we could not determine whether continuous monitoring would have produced similar effects at 25 °C. In addition, only one chamber or incubator was used for each temperature treatment, meaning that temperature effects cannot be separated from possible chamber effects. The cohort-rearing experiment included only five replicate containers per temperature, and developmental time also varied among the four camera platforms used in Experiment 2. Finally, individual molts could not be assigned reliably under cohort-rearing conditions, so Experiment 3 could not determine whether rearing condition affected the number of instars completed. Developmental-time analyses included only individuals that reached adulthood, although the proportion reaching adulthood differed among treatments. The exact date of loss was unknown for eight individuals, and three individuals in Experiment 2 were still developing when the study ended. In addition, we measured development only from the first instar to adulthood. Generation time could not be estimated because preoviposition duration and oothecal incubation time were not recorded. Mold-related losses in Experiment 1 were nearly identical in absolute terms at the two temperatures (14.0% of all individuals at 25 °C and 14.4% at 30 °C); they represented a larger share of losses at 25 °C only because other loss types were less frequent there. If mold losses were nevertheless non-random with respect to developmental rate, the surviving sample could be biased accordingly, and all developmental-time analyses remain conditional on reaching adulthood.

Neither the solitary- nor cohort-rearing protocol should be assumed to represent field conditions. Field populations experience fluctuating temperatures and humidity, complex shelter conditions, mixed-age aggregations, and repeated exposure to environmental microorganisms. The developmental estimates reported here should therefore be considered specific to the protocols used and validated under field conditions before being applied to predictions of population growth or decisions about monitoring and control.

## 5. Conclusions

This study provides the first instar-specific stadium-duration and morphometric series for *P. lateralis* and demonstrates that developmental estimates can vary substantially among rearing protocols. Solitary-reared individuals completed 7–11 instars and required approximately 182–225 d to reach adulthood, whereas median developmental time under cohort-rearing conditions was 79 d at 25 °C and 76 d at 30 °C.

Because molts were not recorded under cohort-rearing conditions, the relationship between rearing protocol and instar number could not be determined. Degree-day totals calculated using a 15 °C base were similar between cohort-rearing temperatures but differed between solitary-rearing temperatures, indicating that a single DD_15_ constant did not adequately describe development under all conditions. Additional independently replicated temperatures are needed to estimate the lower developmental threshold and develop a validated thermal model.

Overall, these findings show that rearing conditions must be considered when applying laboratory-derived life-history estimates to field populations or comparing results among varying laboratory colonies.

## Figures and Tables

**Table 1:** Developmental time from first instar to adulthood under solitary, photographically monitored solitary, and cohort-rearing conditions. Values are mean ± SEM and range in days and degree-days, calculated using an assumed lower developmental threshold of 15 °C.

Rearing	Sex	Temp (°C)	*n*	Mean ± SEM (Days)	Range (Days)	Mean ± SEM (DD)	Range (DD)
Solitary	Female	25	83	225.0 ± 2.07	188–282	2250 ± 20.7	1880–2820
Solitary	Female	30	76	190.2 ± 2.13	141–222	2853 ± 31.9	2115–3330
Solitary	Male	25	93	219.8 ± 1.45	184–259	2198 ± 14.5	1840–2590
Solitary	Male	30	84	181.5 ± 2.51	122–227	2722 ± 37.6	1830–3405
Solitary-video	Female	30	26	255.3 ± 13.24	114–356	3829 ± 198.6	1710–5340
Solitary-video	Male	30	26	221.6 ± 13.94	108–315	3324 ± 209.1	1620–4725
Cohort	Female	25	114	112.4 ± 9.20	64–344	1124.0 ± 92.0	640–3440
Cohort	Female	30	112	77.6 ± 1.30	51–121	1163.7 ± 19.5	765–1815
Cohort	Male	25	112	120.2 ± 7.43	63–330	1201.7 ± 74.3	630–3300
Cohort	Male	30	109	76.5 ± 2.66	50–118	1147.2 ± 39.9	750–1770

**Table 2:** Pairwise comparisons of developmental time to adulthood. A presents Mann–Whitney U comparisons within Experiment 1. B presents comparisons between solitary (Experiment 1) and cohort (Experiment 3) rearing. Adjusted p values use Bonferroni correction within each panel (*** *p* < 0.001; ** *p* < 0.01; ns = not significant).

A. Solitary Rearing (Experiment 1)							
Group 1	Group 2	U	*p*	Adjusted *p*	*r*	Effect Size	Significance
Solitary male 25 °C	Solitary male 30 °C	7302.5	1.90 × 10^−23^	7.60 × 10^−23^	−0.870	Large	***
Solitary female 25 °C	Solitary female 30 °C	5796.5	8.13 × 10^−20^	3.25 × 10^−19^	−0.838	Large	***
Solitary male 25 °C	Solitary female 25 °C	3334.0	0.120	0.480	0.136	Small	ns
Solitary male 30 °C	Solitary female 30 °C	2508.0	0.0195	0.078	0.214	Small	ns
**B. Solitary Rearing (Experiment 1) Versus Cohort Rearing (Experiment 3)**							
**Temp**	**Sex**	**Solitary *n***	**Cohort *k***	**Solitary: Cohort Ratio**	**95% CI**	**Adjusted *p***	**Significance**
25 °C	Male	93	5	1.84	1.54–2.21	0.0027	**
25 °C	Female	83	5	2.02	1.63–2.51	0.0031	**
30 °C	Male	84	5	2.36	2.14–2.60	<0.001	***
30 °C	Female	76	5	2.44	2.33–2.56	<0.001	***

Note: Ratios are mean developmental time under solitary rearing divided by that under cohort rearing; adjusted *p* values are corrected across the four rearing-condition contrasts. Rank-based analyses using the same experimental units are shown in Figure S4.

**Table 3:** Number of solitarily reared cockroaches reaching adulthood after 7–10 instars at 25 and 30 °C (Experiment 1), with associated natural death, mold-related loss, and escape counts.

Instars	Number of Individuals	
	25 °C	30 °C
7	1	0
8	31	12
9	85	80
10	59	68
Total adults	176	160
Natural death	21	46
Death because of mold	35	36
Escaped	18	8
Total sample	250	250

**Table 4:** Mean stadium duration at each instar under solitary rearing at 25 and 30 °C (Experiment 1). Values are mean ± SEM in days and degree-days. All recorded stadia were included. Significance levels are defined as follows: ns = non-significant (*p* > 0.05), * = *p* < 0.05, and *** = *p* < 0.001.

25 °C					
Instar	*n*	Mean ± SEM (Days)	Mean ± SEM (DD)	*p* (Bonferroni)	Significance
First	250	10.01 ± 0.09	100.1 ± 0.91	6.4 × 10^−55^	***
Second	229	12.76 ± 0.20	127.6 ± 1.98	1.6 × 10^−51^	***
Third	209	16.21 ± 0.34	162.1 ± 3.37	7.1 × 10^−35^	***
Fourth	200	20.42 ± 0.59	204.2 ± 5.90	5.0 × 10^−19^	***
Fifth	184	28.91 ± 0.84	289.1 ± 8.43	4.8 × 10^−7^	***
Sixth	180	33.22 ± 0.78	332.2 ± 7.77	2.4 × 10^−15^	***
Seventh	179	33.45 ± 0.75	334.5 ± 7.49	2.9 × 10^−8^	***
Eighth	177	31.55 ± 0.84	315.5 ± 8.43	1.000	ns
Ninth	146	30.92 ± 0.74	309.2 ± 7.44	0.033	*
Tenth	58	33.76 ± 1.28	337.6 ± 12.83	0.093	ns
**30 °C**					
**Instar**	***n***	**Mean ± SEM (Days)**	**Mean ± SEM (DD)**	***p* (Bonferroni)**	**Significance**
First	239	7.77 ± 0.07	116.6 ± 1.12	6.4 × 10^−55^	***
Second	222	8.96 ± 0.11	134.5 ± 1.67	1.6 × 10^−51^	***
Third	201	11.38 ± 0.30	170.7 ± 4.47	7.1 × 10^−35^	***
Fourth	178	14.41 ± 0.47	216.2 ± 7.05	5.0 × 10^−19^	***
Fifth	171	23.04 ± 0.69	345.6 ± 10.31	4.8 × 10^−7^	***
Sixth	166	24.39 ± 0.71	365.9 ± 10.62	2.4 × 10^−15^	***
Seventh	160	27.31 ± 0.70	409.6 ± 10.45	2.9 × 10^−8^	***
Eighth	160	30.21 ± 0.69	453.2 ± 10.32	1.000	ns
Ninth	148	27.97 ± 0.73	419.5 ± 10.97	0.033	*
Tenth	68	29.22 ± 1.14	438.3 ± 17.07	0.093	ns

**Table 5:** Morphometric measurements (mean ± SEM, range; mm) of head width, pronotum width, pronotum length, and body length by instar and sex for *P. lateralis* reared at 25 and 30 °C (Experiment 1). Lowercase superscript letters summarize Holm-adjusted developmental-stage comparisons from the ART/emmeans analysis within each temperature; asterisks mark Holm-adjusted between-temperature Welch contrasts within the same stage and trait.

Instars	*n*	Head Width		Pronotum Width		Pronotum Length		Body Length	
		Mean ± SEM	Range	Mean ± SEM	Range	Mean ± SEM	Range	Mean ± SEM	Range
25 °C									
1	50	1.16 ± 0.01 ^a^	1.02–1.30	1.66 ± 0.01 ^a^	1.38–1.81	1.17 ± 0.01 ^a^	1.01–1.29	4.76 ± 0.04 ^a^	4.18–5.29
2	50	1.38 ± 0.01 ^b^	1.09–1.60	2.25 ± 0.02 ^b^	1.79–2.65	1.47 ± 0.02 ^b^	1.15–1.93	6.04 ± 0.06 ^b^	4.99–7.38
3	50	1.54 ± 0.02 ^c^	1.18–1.80	2.70 ± 0.03 ^c^	2.15–3.03	1.82 ± 0.03 ^c^	1.21–2.60	7.28 ± 0.08 ^c^	6.19–8.43
4	50	1.85 ± 0.02 ^d^	1.54–2.16	3.32 ± 0.03 ^d^	2.63–3.76	2.16 ± 0.03 ^d^	1.52–2.69	9.07 ± 0.09 ^d^	7.63–10.90
5	50	2.07 ± 0.02 ^e^	1.70–2.32	3.76 ± 0.04 ^e^	3.08–4.23	2.49 ± 0.03 ^e^	1.91–2.85	10.77 ± 0.12 ^e^	8.48–12.50
6	50	2.40 ± 0.03 ^f^	1.93–2.90	4.37 ± 0.04 ^f^	3.61–4.90	2.97 ± 0.04 ^f^	2.30–3.45	12.62 ± 0.13 ^f^	10.70–15.20
7	50	2.83 ± 0.03 ^g^	2.09–3.34	5.01 ± 0.06 ^g^	4.07–5.74	3.37 ± 0.05 ^g^	2.53–4.14	14.77 ± 0.20 ^g^	11.60–17.40
8	50	3.14 ± 0.03 ^h^	2.37–3.59	5.76 ± 0.07 ^h^	4.32–6.83	3.72 ± 0.05 ^h^	2.71–4.58	17.56 ± 0.23 ^h^	14.10–22.40
9	50	3.42 ± 0.03 ^i^	2.90–3.88	6.45 ± 0.08 ^i^	4.41–7.17	4.22 ± 0.07 ^i^	3.04–5.10	20.29 ± 0.27 ^i^	14.00–24.00
10	50	3.67 ± 0.04 ^j^	3.13–4.30	6.92 ± 0.07 ^j^	5.34–7.82	4.66 ± 0.08 ^j^	3.68–6.22	22.12 ± 0.17 ^j^	19.10–25.63
Male	25	3.70 ± 0.03 ^j^	3.47–3.93	6.68 ± 0.05 ^k^	6.25–7.17	4.46 ± 0.08 ^j^	3.68–5.28	26.04 ± 0.29 ^k^	20.90–27.92
Female	25	3.92 ± 0.05 ^k^	3.49–4.34	7.88 ± 0.05 ^l^	7.35–8.39	5.25 ± 0.10 ^k^	4.16–6.16	25.29 ± 0.31 ^k^	23.10–28.50
**30 °C**									
1	50	1.15 ± 0.01 ^a^	0.83–1.28	1.61 ± 0.02 ^a^	1.16–1.80	1.13 ± 0.01 ^a^	0.73–1.31	4.76 ± 0.05 ^a^	3.32–5.58
2	50	1.34 ± 0.01 ^b^	1.14–1.49	2.13 ± 0.02 ^b^**	1.76–2.36	1.42 ± 0.02 ^b^	1.14–1.66	5.85 ± 0.06 ^b^	4.31–6.77
3	50	1.52 ± 0.02 ^c^	0.99–1.84	2.60 ± 0.03 ^c^	2.04–2.97	1.72 ± 0.03 ^c^	1.34–2.02	7.20 ± 0.10 ^c^	5.52–8.79
4	50	1.71 ± 0.02 ^d^***	1.27–1.98	2.93 ± 0.07 ^d^***	1.85–3.55	2.11 ± 0.03 ^d^	1.75–2.51	8.96 ± 0.10 ^d^	7.70–10.20
5	50	2.06 ± 0.03 ^e^	1.65–3.07	3.53 ± 0.06 ^e^	2.00–4.07	2.47 ± 0.05 ^e^	1.46–3.76	10.62 ± 0.13 ^e^	7.80–12.90
6	50	2.36 ± 0.03 ^f^	1.98–2.76	4.18 ± 0.05 ^f^	3.12–4.94	2.96 ± 0.03 ^f^	2.53–3.53	12.91 ± 0.14 ^f^	10.40–15.10
7	50	2.68 ± 0.03 ^g^	2.16–3.29	4.49 ± 0.09 ^f^***	3.16–5.51	3.39 ± 0.03 ^g^	2.80–3.81	14.62 ± 0.12 ^g^	12.70–16.20
8	50	3.00 ± 0.03 ^h^	2.28–3.79	5.36 ± 0.05 ^g^***	4.36–5.93	3.84 ± 0.05 ^h^	3.15–4.34	16.40 ± 0.13 ^h^**	13.90–18.10
9	50	3.34 ± 0.03 ^i^	2.87–3.77	6.39 ± 0.05 ^h^	5.40–7.01	4.27 ± 0.07 ^i^	3.26–4.99	19.17 ± 0.21 ^i^*	16.50–22.80
10	50	3.72 ± 0.04 ^j^	3.27–4.68	6.96 ± 0.10 ^hi^	4.02–7.86	4.71 ± 0.10 ^j^	3.33–6.18	22.36 ± 0.26 ^j^	18.90–26.43
Male	25	3.82 ± 0.04 ^j^	3.36–4.25	6.80 ± 0.08 ^i^	5.91–7.52	4.64 ± 0.08 ^j^	3.75–5.26	26.24 ± 0.37 ^k^	19.90–28.60
Female	25	4.10 ± 0.06 ^k^	3.24–4.43	8.13 ± 0.09 ^j^	7.08–8.92	5.36 ± 0.13 ^k^	4.32–6.57	25.27 ± 0.68 ^l^	15.10–30.20

Note: *n* denotes the number of individuals selected for morphometric measurement at each developmental stage; trait-specific sample sizes can be slightly smaller when an individual measurement was unavailable. Exact trait-specific sample sizes for the between-temperature tests are reported in Table S3. Different lowercase superscript letters indicate significant differences among developmental stages within the same temperature (ART/emmeans, Holm-adjusted; α = 0.05). Asterisks beside 30 °C values indicate a significant difference from the corresponding 25 °C value for that stage and trait (Welch *t*-test, Holm-corrected across 48 comparisons): * *p* < 0.05; ** *p* < 0.01; *** *p* < 0.001. Full between-temperature statistics are provided in Table S3. Because measurement ranges overlap among adjacent late instars, the table should not be used as a stand-alone diagnostic for instar assignment.

**Table 6:** Comparison of stadium duration between Experiments 1 and 2 at 30 °C. Ratios greater than 1 indicate longer stadium duration in Experiment 2. Ratios, 95% confidence intervals, and adjusted *p* values are direct contrasts from the mixed-effects model, with Bonferroni correction across the ten instars. Only individuals that reached adulthood were included. Significance levels are defined as follows: ns = non-significant (*p* > 0.05), * = *p* < 0.05, and *** = *p* < 0.001.

Instar	Experiment 1 *n*	Experiment 1 Mean ± SEM	Experiment 2 *n*	Experiment 2 Mean ± SEM	Ratio Exp. 2:Exp. 1	Adjusted *p*	Significance
1	160	7.71 ± 0.09	52	8.31 ± 0.17	1.08 (0.97–1.19)	1.000	ns
2	160	9.00 ± 0.14	52	11.40 ± 0.36	1.25 (1.13–1.39)	<0.001	***
3	160	11.22 ± 0.34	52	14.92 ± 0.67	1.33 (1.20–1.48)	<0.001	***
4	160	14.41 ± 0.51	52	20.02 ± 0.94	1.43 (1.29–1.59)	<0.001	***
5	160	23.07 ± 0.71	52	23.33 ± 1.41	0.97 (0.87–1.07)	1.000	ns
6	160	24.39 ± 0.73	52	29.08 ± 2.29	1.15 (1.03–1.27)	0.103	ns
7	160	27.31 ± 0.70	52	34.15 ± 2.19	1.17 (1.06–1.30)	0.030	*
8	160	30.21 ± 0.69	52	37.21 ± 2.13	1.17 (1.05–1.30)	0.036	*
9	148	27.97 ± 0.73	45	37.93 ± 2.34	1.29 (1.15–1.44)	<0.001	***
10	68	29.22 ± 1.14	28	36.64 ± 3.06	1.19 (1.02–1.38)	0.226	ns
11	0	—	15	31.73 ± 2.89	—	—	—

Note: Instar 11 occurred only in Experiment 2 and could not be compared.

## Data Availability

The data presented in this study are available on request from the corresponding author.

## Supplementary Materials

The following supporting information can be downloaded at: https://www.mdpi.com/article/10.3390/insects17090948/s1, Figure S1: experimental setup for solitary rearing; Figure S2: photographic monitoring system; Figure S3: reciprocal of mean development time under solitary and cohort rearing; Figure S4: signed rank-biserial effect sizes; Table S1: stadium duration by instar and temperature (Experiment 1); Table S2: Wilcoxon rank-sum comparisons of stadium duration between Experiments 1 and 2; Table S3: between-temperature comparisons of morphometric traits.
