# Supplementary material for: Development and Morphometrics of the Turkestan Cockroach (Periplaneta lateralis) Under Solitary and Cohort Rearing at 25 and 30 °C
Source: Insects. 2026 Sep 11;17(9):948. doi: 10.3390/insects17090948 (PMC13607706; doi:10.3390/insects17090948)
Supplement: Supplementary file 1 [file insects-17-00948-s001.zip › insects-4498330-supplementary.pdf]

## Supplemental figures and tables

The following are available as supplementary material. Figure S1: experimental setup for solitary rearing; Figure S2: photographic monitoring system; Figure S3: reciprocal of mean development time under solitary and cohort rearing; Figure S4: signed rank-biserial effect sizes; Table S1: stadium duration by instar and temperature (Experiment 1); Table S2: Wilcoxon rank-sum comparisons of stadium duration between Experiments 1 and 2; Table S3: between-temperature comparisons of morphometric traits.

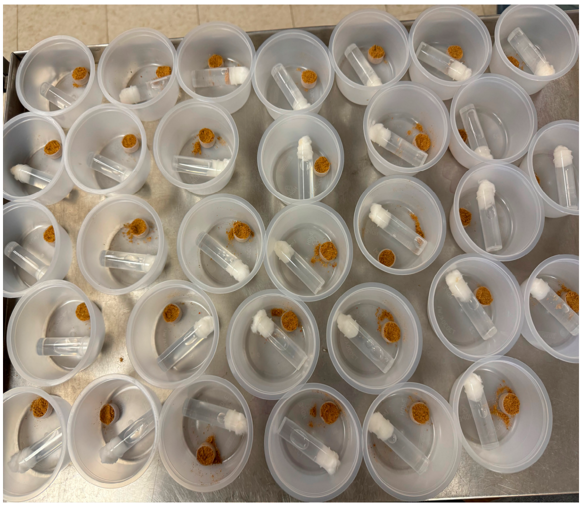

**Figure S1.** Experimental setup used to evaluate cockroach life-history traits under solitary rearing conditions. Cockroaches were individually housed in tapered round plastic cups containing ad libitum food and water. Cups were stacked within environmental chambers maintained at either 25 or 30 °C.

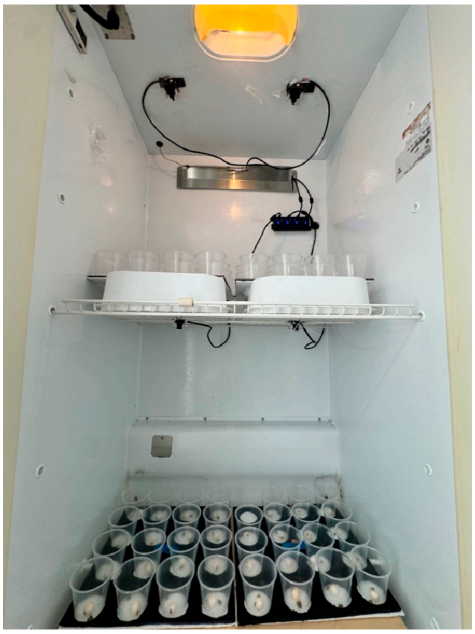

**Figure S2.** Photographic monitoring system used to record individual cockroach development. Four 1080p day-and-night-vision Arducam USB cameras were mounted 55 cm above 31 × 31 cm platforms, with each camera monitoring 16 evenly spaced containers. Images were captured at 30-min intervals and stored on a laptop.

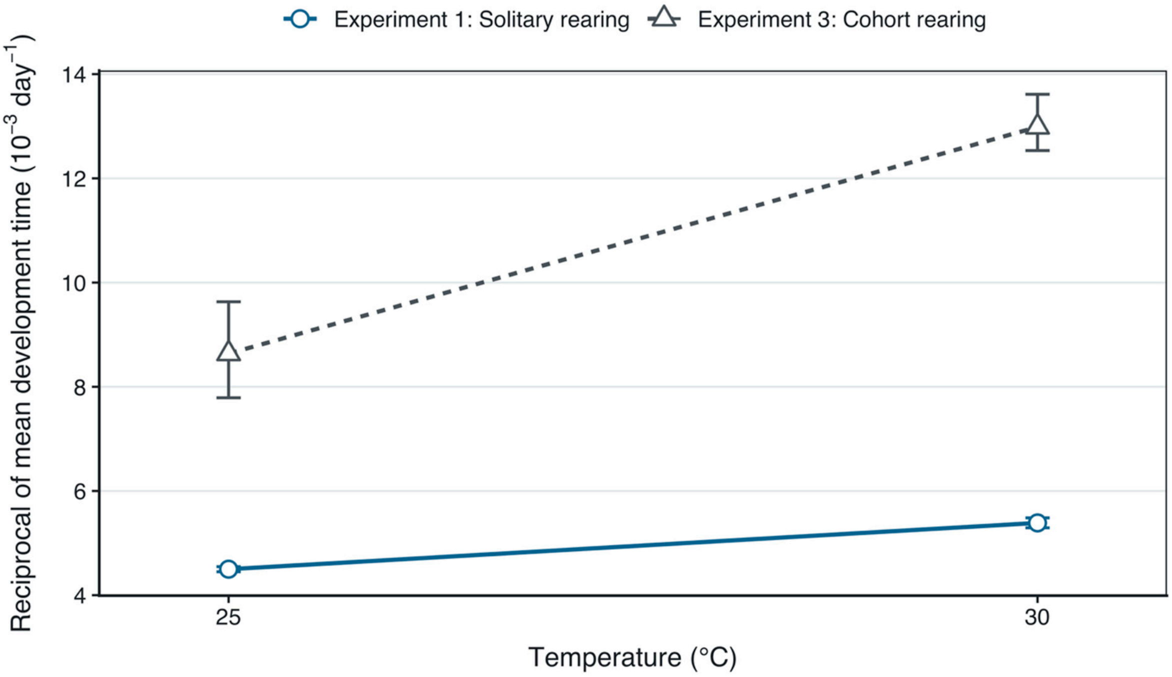


**Figure S3.** Developmental rate under solitary and cohort rearing at 25 °C and 30 °C. Developmental rate was calculated as the reciprocal of mean developmental time. Error bars show bootstrap 95% confidence intervals. Connecting lines join the two observed temperatures and do not represent fitted developmental models.

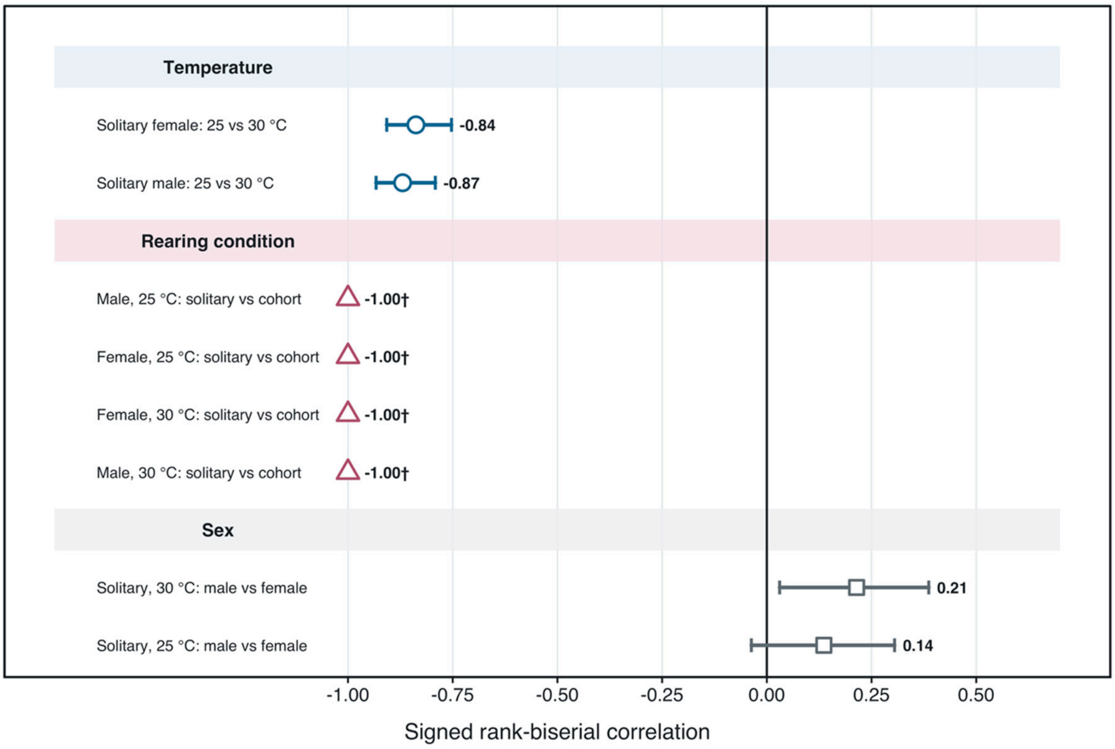


**Figure S4:** Rank-biserial effect sizes for selected comparisons of developmental time. Points show signed rank-biserial correlations, and error bars show bootstrap 95% confidence intervals. Dashed lines indicate medium and large effect-size thresholds. For solitary-versus-cohort comparisons, individual cockroaches were the experimental units under solitary rearing and container means were the experimental units under cohort rearing. A dagger marks complete separation of the compared distributions.

**Table S1.** Median stadium duration and interquartile range for solitarily reared cockroaches at 25 °C and 30 °C. Only individuals that reached adulthood were included. These values are plotted in Figure 3.

| Temperature (°C) | Instar | n | Median (days) | Q1 | Q3 |
|---|---|---|---|---|---|
| 25 | 1 | 176 | 10 | 9 | 10 |
| 25 | 2 | 176 | 12 | 11 | 13 |
| 25 | 3 | 176 | 15 | 13 | 18 |
| 25 | 4 | 176 | 18 | 15 | 23.25 |
| 25 | 5 | 176 | 26 | 20.75 | 33.25 |
| 25 | 6 | 176 | 32 | 25 | 39 |
| 25 | 7 | 176 | 32 | 26 | 39 |
| 25 | 8 | 175 | 28 | 24 | 37 |
| 25 | 9 | 144 | 29 | 24.75 | 36 |
| 25 | 10 | 58 | 35 | 24.25 | 40 |
| 30 | 1 | 160 | 8 | 7 | 8 |
| 30 | 2 | 160 | 9 | 8 | 10 |
| 30 | 3 | 160 | 10 | 9 | 12 |
| 30 | 4 | 160 | 12 | 10 | 17 |
| 30 | 5 | 160 | 22 | 17 | 26 |
| 30 | 6 | 160 | 22 | 17 | 30 |
| 30 | 7 | 160 | 25.5 | 21 | 32 |
| 30 | 8 | 160 | 28 | 23 | 37 |
| 30 | 9 | 148 | 27 | 21 | 32 |
| 30 | 10 | 68 | 28 | 21 | 37 |

**Table S2.** Wilcoxon rank-sum comparisons of stadium duration between Experiments 1 and 2 at 30 °C. Tests were conducted separately for each instar as a sensitivity analysis for the mixed-effects model. Adjusted p values use Bonferroni correction across the ten instars. Only individuals that reached adulthood were included.

| Instar | n (Experiment 1) | n (Experiment 2) | p | p (Bonferroni) | Significance |
|---|---|---|---|---|---|
| 1 | 160 | 52 | 3.23e-04 | 0.003 | ** |
| 2 | 160 | 52 | 6.30e-10 | 6.30e-09 | *** |
| 3 | 160 | 52 | 3.97e-11 | 3.97e-10 | *** |
| 4 | 160 | 52 | 2.24e-10 | 2.24e-09 | *** |
| 5 | 160 | 52 | 0.600 | 1.000 | ns |
| 6 | 160 | 52 | 0.068 | 0.676 | ns |
| 7 | 160 | 52 | 0.011 | 0.110 | ns |
| 8 | 160 | 52 | 0.006 | 0.061 | ns |
| 9 | 148 | 45 | 5.65e-05 | 5.65e-04 | *** |
| 10 | 68 | 28 | 0.027 | 0.271 | ns |

**Table S3.** Between-temperature comparisons of morphometric traits of Periplaneta lateralis reared solitarily at 25 and 30 °C (Experiment 1). Traits were compared between temperatures within each instar and within each adult sex using Welch t-tests; p values are Holm-corrected across the 48 comparisons. Significant results are marked in Table 5.
* $p < 0.05$; ** $p < 0.01$; *** $p < 0.001$; ns = not significant after Holm correction. Means are in mm. Comparisons are between temperatures within an instar; the table does not compare instars with one another.

| **Instar** | **Trait** | **n (25 °C)** | **n (30 °C)** | **Mean 25 °C** | **Mean 30 °C** | **t** | **p (Holm)** | |
|---|---|---|---|---|---|---|---|---|
| First | Head width | 50 | 50 | 1.16 | 1.15 | 0.84 | 1.000 | ns |
| First | Pronotum width | 50 | 50 | 1.66 | 1.61 | 2.36 | 0.663 | ns |
| First | Pronotum length | 50 | 50 | 1.17 | 1.13 | 2.59 | 0.396 | ns |
| First | Body length | 50 | 50 | 4.76 | 4.76 | -0.07 | 1.000 | ns |
| Second | Head width | 50 | 50 | 1.38 | 1.34 | 2.62 | 0.371 | ns |
| Second | Pronotum width | 50 | 50 | 2.25 | 2.13 | 4.00 | 0.005 | ** |
| Second | Pronotum length | 50 | 50 | 1.47 | 1.42 | 2.10 | 1.000 | ns |
| Second | Body length | 50 | 50 | 6.04 | 5.85 | 2.19 | 0.928 | ns |
| Third | Head width | 50 | 50 | 1.54 | 1.52 | 0.83 | 1.000 | ns |
| Third | Pronotum width | 50 | 50 | 2.70 | 2.60 | 2.50 | 0.481 | ns |
| Third | Pronotum length | 50 | 50 | 1.82 | 1.72 | 2.67 | 0.328 | ns |
| Third | Body length | 50 | 50 | 7.28 | 7.20 | 0.61 | 1.000 | ns |
| Fourth | Head width | 49 | 50 | 1.85 | 1.71 | 4.79 | <0.001 | *** |
| Fourth | Pronotum width | 48 | 50 | 3.31 | 2.93 | 4.75 | <0.001 | *** |
| Fourth | Pronotum length | 49 | 50 | 2.16 | 2.11 | 1.28 | 1.000 | ns |
| Fourth | Body length | 49 | 50 | 9.07 | 8.96 | 0.87 | 1.000 | ns |
| Fifth | Head width | 49 | 49 | 2.08 | 2.06 | 0.38 | 1.000 | ns |
| Fifth | Pronotum width | 49 | 49 | 3.76 | 3.52 | 3.24 | 0.072 | ns |
| Fifth | Pronotum length | 49 | 49 | 2.49 | 2.46 | 0.57 | 1.000 | ns |
| Fifth | Body length | 49 | 49 | 10.76 | 10.60 | 0.95 | 1.000 | ns |
| Sixth | Head width | 49 | 49 | 2.40 | 2.36 | 0.95 | 1.000 | ns |
| Sixth | Pronotum width | 49 | 49 | 4.38 | 4.18 | 3.00 | 0.134 | ns |
| Sixth | Pronotum length | 49 | 49 | 2.97 | 2.96 | 0.18 | 1.000 | ns |
| Sixth | Body length | 49 | 49 | 12.64 | 12.89 | -1.32 | 1.000 | ns |
| Seventh | Head width | 49 | 49 | 2.83 | 2.68 | 3.16 | 0.084 | ns |
| Seventh | Pronotum width | 49 | 49 | 5.02 | 4.49 | 4.87 | <0.001 | *** |
| Seventh | Pronotum length | 49 | 49 | 3.37 | 3.39 | -0.41 | 1.000 | ns |
| Seventh | Body length | 49 | 49 | 14.80 | 14.63 | 0.71 | 1.000 | ns |
| Eighth | Head width | 50 | 49 | 3.14 | 3.00 | 3.00 | 0.134 | ns |

| | | | | | | | | |
|---|---|---|---|---|---|---|---|---|
| Eighth | Pronotum width | 50 | 49 | 5.76 | 5.35 | 4.80 | <0.001 | *** |
| Eighth | Pronotum length | 50 | 49 | 3.72 | 3.84 | -1.69 | 1.000 | ns |
| Eighth | Body length | 50 | 49 | 17.56 | 16.39 | 4.43 | 0.001 | ** |
| Ninth | Head width | 49 | 49 | 3.43 | 3.33 | 2.13 | 1.000 | ns |
| Ninth | Pronotum width | 49 | 48 | 6.46 | 6.38 | 0.90 | 1.000 | ns |
| Ninth | Pronotum length | 49 | 49 | 4.23 | 4.26 | -0.34 | 1.000 | ns |
| Ninth | Body length | 49 | 49 | 20.33 | 19.17 | 3.36 | 0.048 | * |
| Tenth | Head width | 49 | 50 | 3.67 | 3.72 | -1.02 | 1.000 | ns |
| Tenth | Pronotum width | 49 | 50 | 6.91 | 6.96 | -0.42 | 1.000 | ns |
| Tenth | Pronotum length | 49 | 50 | 4.66 | 4.71 | -0.38 | 1.000 | ns |
| Tenth | Body length | 49 | 50 | 22.13 | 22.36 | -0.75 | 1.000 | ns |
| Adult male | Head width | 24 | 25 | 3.71 | 3.82 | -2.34 | 0.779 | ns |
| Adult male | Pronotum width | 24 | 25 | 6.69 | 6.80 | -1.26 | 1.000 | ns |
| Adult male | Pronotum length | 24 | 25 | 4.47 | 4.64 | -1.50 | 1.000 | ns |
| Adult male | Body length | 24 | 25 | 26.07 | 26.24 | -0.35 | 1.000 | ns |
| Adult female | Head width | 24 | 25 | 3.93 | 4.10 | -2.13 | 1.000 | ns |
| Adult female | Pronotum width | 24 | 25 | 7.89 | 8.13 | -2.29 | 0.853 | ns |
| Adult female | Pronotum length | 24 | 25 | 5.24 | 5.36 | -0.77 | 1.000 | ns |
| Adult female | Body length | 24 | 25 | 25.34 | 25.27 | 0.10 | 1.000 | ns |
